# Embodiment and Psychological Health in Adolescence: 2. Embodiment Profiles and their Association with Psychological Health Among Young Adolescents

**DOI:** 10.17505/jpor.2025.27578

**Published:** 2025-04-01

**Authors:** Lars-Gunnar Lundh, Lo Foster, Daiva Daukantaitė

**Affiliations:** 1 Department of Psychology, Lund University, Lund, Sweden, lars-gunnar.lundh@psy.lu.se ORCID: https://orcid.org/0000-0002-1649-969X; 2 Department of Psychology, Lund University, Lund, Sweden, lo.foster@psy.lu.se ORCID: https://orcid.org/0009-0008-3636-956X; 3 Department of Psychology, Lund University, Lund, Sweden, daiva.daukantaite@psy.lu.se ORCID: https://orcid.org/0000-0002-1994-041X

**Keywords:** embodiment, Embodiment Scale-12 (ES-12), embodiment profiles, adolescence, disordered eating, non-suicidal self-injury, depression, anxiety, cluster analysis, Body for Others

## Abstract

**Background:**

Several researchers have argued that disturbances in embodiment play an important role in the development of psychological health problems among adolescents. The purpose of the present study was to use the 12-item Embodiment Scale (ES-12), with its three subscales Harmonious Body (HB), Disharmonious Body (DB), and Body for Others (BO), (1) to identify subgroups of adolescents with different profiles on the ES-12 scales, and (2) to see how these profiles are associated with patterns of psychological health problems.

**Methods:**

The participants were 530 adolescents with a mean age of 14 years (*SD* = 0.89), who filled out the ES-12 and measures of disordered eating, non-suicidal self-injury (NSSI), anxiety, and depression. Hierarchical cluster analysis was used to divide the sample into (1) subgroups with different profiles of HB, DB and BO and (2) subgroups with different psychological health profiles. Cross-tabulation was used to study associations between different embodiment profiles and different patterns of psychological health problems.

**Results:**

Five different profiles of embodiment were identified: *Strong Embodiment, Average Embodiment, Weak Embodiment, Body for Others, and Low Body Harmony*. Five different psychological health profiles were identified: *Multiple Problems, Multiple Problems without NSSI, Disordered Eating Only, Average Psychological Health,* and a *Healthy* profile. Cross-tabulation showed that individuals with a *Strong Embodiment* profile were over-represented among adolescents in the *Healthy* cluster, and that individuals with the *Weak Embodiment* profile were over-represented in both *Multiple Problems* clusters. Individuals with a *Body for Others* profile were overrepresented among adolescents with a *Disordered Eating Only* profile but not among adolescents with *Multiple Problems* profiles.

**Conclusions:**

These findings align with theoretical frameworks which emphasize the importance of embodiment for the understanding of psychological health problems. At the same time, the results go against theories that attribute a central role to high levels of experienced Body for Others for the development of psychological health problems.

## Introduction

Several researchers (e.g., Fuchs, 2022; Piran & Teall, 2012; Stanghellini et al., 2012, 2019) have argued that disturbances in embodiment are important for the development of psychological health problems among adolescents, such as disordered eating and full-blown eating disorders (EDs). The concept of embodiment, however, contains different aspects and has been analyzed in different ways by different theorists. For example, several different ideas have been suggested for why embodiment disturbances may be centrally involved in eating disorders (e.g., Fuchs, 2022; Legrand, 2010; Lundh & Foster, 2024; Stanghellini et al., 2012, 2019).

In a recent study, Foster et al. (2025) described the development and comprehensive validation of a brief twelve-item Embodiment Scale (*ES-12*) suitable for young adolescents. Factor analysis identified three *ES-12* subscales, which were labeled *Harmonious Body* (HB), *Disharmonious Body* (DB), and *Body for Others* (BO). The HB subscale contains items referring to having a positive relationship to one’s own body (as seen in the items “I am friends with my body”, “I enjoy having the body I have”, and “I feel at home in my body”), and as caring more about bodily feelings than outward appearances (“I care more about how the body feels than how it looks”), and a congruity between inner feelings and outward expression (“My body reflects who I feel I am inside”). The DB subscale, in contrast, contains items expressing a conflictual relationship to one’s body, including discomfort in experiencing bodily feelings (“It is unpleasant to feel what it feels like in my body”), feelings of disconnection from the body (“I can feel separated and disconnected from my body”), and experiencing the body as a kind of hindrance (”My body prevents me from doing what I want (such as playing sports or hanging out with friends)”. Finally, the BO subscale contains items about focusing on how one’s body is seen by others (“I think about how my body looks to others”), and the perceived importance of what others think about one’s bodily appearance (“It is important to me that others do not think I am physically weak” and “It is important to me what other people think about my appearance and physical characteristics”).

Importantly, Foster et al. (2025) found that experienced embodiment, as measured by the three *ES-12* scales, predicted psychological health problems (disordered eating, non-suicidal self-injury, depression and anxiety) beyond that of a measure of body dissatisfaction. This is particularly interesting in view of previous longitudinal research which has found body dissatisfaction to predict the development of disordered eating (e.g., Foster et al., 2024), non-suicidal self-injury (NSSI; Black et al., 2019), and depression (Blundell et al., 2024) during adolescence. In conclusion, Foster et al. (2025) suggested that the *ES-12* may capture aspects of bodily self-experience that may be even more important than body dissatisfaction for the development of various aspects of psychopathology.

Of the three dimensions of embodiment captured by the *ES-12*, the notion of “Body for Others” has been given the most attention by theorists in this field. The importance attached to this dimension, however, varies between different theories. Some theorists (e.g., Piran & Teall, 2012; Stanghellini et al., 2012) attach large importance to this dimension *in itself*, whereas others (e.g., Legrand, 2010, 2011) argue that the degree to which an individual attends to how they are viewed by others may have widely different meaning *depending on other aspects of their bodily self-experience*. This is a research topic where a person-oriented approach is especially well suited. As Bergman and Andersson (2010), formulates it, in a person-oriented approach “the variable values achieve their importance as parts of an indivisible pattern; they have no separate status; it is the profile of scores that matters” (p. 157).

### Body for Others – Contrasting Theoretical Perspectives and Empirical Findings

The focus on *Body for Others* as the most important dimension of bodily self-experience for some forms of psychopathology is most clearly expressed by Stanghellini et al. (2012, 2019). Influenced by Sartre (1943/1958), Stanghellini et al. (2012) formulated the hypothesis “that persons with EDs experience their own body first and foremost as an object being looked at by another” (p. 148). As summarized by Stanghellini et al. (2019):

There are theoretical as well as clinical reasons to consider abnormal eating behaviors as epiphenomena of a more profound disorder of lived corporeality and self-identity. Especially one dimension – the lived body-for-others as described by J.-P. Sartre – seems to represent the core concept to grasp the anomalies of lived corporeality in ED patients. (Stanghellini et al., 2019, p. 138)

Writing about girls’ and women’s development in general, Piran and Teall (2012) similarly attach a clear and unambiguous role to the how the body is experienced by others. They assume that the internalization of the external gaze is disruptive to a woman’s experience of embodiment even when it leads to a “positive” body image, because “it implies an objectified perspective of the body” (Piran & Teall, 2012, p. 175).

Legrand (2010) presents a more complex picture of the role of embodiment in eating disorders. In her multi-dimensional perspective, the objectification of the female body represents one of several important dimensions of bodily self-experience. Other equally important dimensions are of a more subjective kind and involves “the body as a bearer of sensations and body-ownership” (p. 732). As she sees it, pathology occurs when there is an *imbalance* of these dimensions: “we are normally conscious of our body as an intertwinement of subjective and physical dimensions: bodily self-consciousness is normally multidimensional. The imbalance of these dimensions relative to each other is pathological” (p. 726). In other words, the experience of one’s body as an object for others to look at is not seen as the core problem. If this experience is interweaved with positive experiences of the subjectively felt body, Legrand argues, there is no problematic self-objectification. As she summarizes it,

experiencing one’s body’s physicality can occur in circumstances where the subjectivity of the person is preserved, respected or even enhanced; while it can also occur in circumstances where the person experiences her subjectivity to be spoiled, reduced, neglected, alienated. The process of objectification is thus damaging not because it brings physical dimensions into bodily self-consciousness, since these dimensions are part of the normal picture. Rather, it is damaging if it is disruptive of subjective dimensions, thereby being disruptive of the integrity of bodily self-consciousness. (Legrand, 2010, p. 730)

Legrand’s analysis is of special importance in the present context, as it points to the importance of *patterns* of embodiment rather than an elevated level of the single dimension of Body for Others. Her reasoning implies that *one and the same level of “Body for Others”-experience may have widely different meaning*, depending on the individual person’s level of more subjective dimensions of bodily experience.

Interestingly, Legrand (2010) uses the notions of “tension” and “balance” in a way that is partly similar to the notions of harmonious and disharmonious embodiment, as measured by the *ES-12*. She pictures anorexia nervosa as involving “a tension between several dimensions of bodily self-consciousness (subjectivity and physicality), dimensions which are normally integrated to each other in a balanced manner” (Legrand, 2010, p. 735). Her way of contrasting “tension between several dimensions of bodily self-consciousness” versus these dimensions being “integrated to each other in a balanced manner” converges with the present conceptualization of embodiment as being harmonious or disharmonious. Interestingly, it also suggests the possibility that strong feelings of “Body for Others” need not be problematic in themselves, but only when occurring in combination with experiences of low bodily harmony and/or high bodily disharmony.

In summary, these theories apparently entail different predictions concerning the importance of the dimension of *Body for Others*, that is, the degree to which the individual attaches value to how their body appears to others. Although there is some previous research (e.g., Cascino et al. 2019; Stanghellini et al., 2012) on the importance of the dimension *Body for Others*, this is only from a variable-oriented perspective, which focuses on analyzing relationships between *Body for Others* and other variables. To test their hypothesis, Stanghellini et al. (2012) developed a self-report questionnaire, the IDentity and EAting disorders (IDEA) with four factors, of which factor one was labelled *Feeling oneself only through the gaze of the other and defining oneself only through the evaluation of the other* (shortened GEO). They then studied the associations between these factors and ED symptoms in a clinical sample of patients with eating disorders. Although their results showed that the total IDEA score was significantly associated with degree of ED pathology, the GEO factor was only found to be associated with one aspect of ED pathology: shape concern.

Moreover, in a network analysis study of the associations between the IDEA factors and ED symptoms in a clinical sample of patients with anorexia nervosa, Cascino et al. (2019) found that only two of the *other* IDEA factors showed the expected kind of association with ED symptoms, whereas the GEO factor (i.e., the *Body for Others* factor) did not. In other words, even from a variable-oriented perspective the support for Stanghellini et al.’s hypothesis is meagre.

Results pointing in a similar direction were found in Foster et al.’s (2025) study of young adolescents, where the subscale *Body for Others* tended to show weaker correlations with psychological health problems (*r*s ranging from .22 to .51) than the subscales *Harmonious Body* (which showed negative correlations ranging from -.45 to -.66 with these psychological health problems) and *Disharmonious Body* (which showed positive correlations ranging from .42 to .60).

Variable-oriented analyses such as these, however, do not take into account the possibility that the same level of *Body for Others* may have different meaning, depending on the pattern of values which it is part of. There is therefore reason to approach this topic also with person-oriented methods, to see if this can help to illuminate the phenomena that are involved. In other words: Could it be that elevated levels of *Body for Others* are found in different subgroups of adolescents with different embodiment profiles? And if so, are these profiles differently associated with psychological health problems?

### Harmonious and Disharmonious Bodily Self-Experience

Analogous questions can also be asked about the other two dimensions measured by the *ES-12*: Are elevated levels of HB and/or DB found in different subgroups of adolescents with different embodiment profiles? And if so, are these profiles differently associated with psychological health problems? Here, however, there is little previous theorization or empirical evidence to formulate more explicit research questions.

Fuchs’ (2022) reasoning is compatible with a person-oriented approach and a focus on interactions between different factors. Although he describes the experience of the other’s gaze “as the major trigger of anorexia” (p. 111), and as “favored by prevailing ideals of beauty and the ‘marketing’ of the body” (p. 111), he sees this merely as one among several changes that occur during adolescence. Among other important developments that occur are (1) objective changes in the body (e.g., girls’ development of more feminine body forms) that “entail new and unfamiliar forms of embodiment” (p. 111); (2) changes in the subjectively felt body, in the form of emerging sexual drives and desires, “which can be experienced as promising, but also as irritating or even threatening” (p. 111); and (3) “a central existential transition… from childhood, which can be experienced with feelings of loss, sadness, abandonment and loneliness” (p. 111). Altogether, this points in the direction of the two other factors in the *ES-12*: HB and DB. Bodily changes, whether they refer to the objective body or the body as felt from within, must be accommodated in some way. If the bodily changes are accepted and well adapted to, it might be expressed in high scores on HB and low scores on DB. On the contrary, if the individual finds it difficult to accept their bodily changes, it might be seen in high scores on DB and low scores on HB.

Piran’s (2018) developmental theory of embodiment also involves a differentiation between positive embodiment and negative embodiment, which is partly similar to the *ES-12* factors HB and DB. As Piran (2018) defines positive embodiment, it includes positive connections to one’s body, experiences of bodily comfort, embodied agency and passion, and attuned self-care. Conversely, her definition of negative embodiment includes disrupted connections to the body, bodily discomfort, restricted agency and passion, and self-neglect. She does not, however, discuss the possibility that there might be embodiment profiles which combine positive and negative embodiment in different ways.

It should be noted that, although Foster et al. (2025) found that HB and DB are strongly correlated (*r* = -.60), their results also showed that HB and DB are partly independent dimensions, with a combined factor of HB and DB yielding a worse fit to the data compared to analyzing the three factors separately. This means that there may well be subgroups of individuals who score low on HB without scoring high on DB, and vice versa. For example, it is quite possible that some individuals report a low level of HB, as seen in low scores on items about having a positive relationship to one’s body (e.g., “I enjoy the body I have” and “My body reflects who I feel I am inside”) without having conflictual feelings to the body of the kind that is asked for in the DB items (e.g., “It is unpleasant to feel what it feels like in my body” and “I can feel separated and disconnected from my body”). It is also quite possible that some individuals may endorse items on both HB and DB, because they tend to alternate between these kinds of experiences.

### The Present Study

The purpose of the present study was to use a person-oriented approach to the study of embodiment and its association with psychological health problems, to see if this could add new information and provide a new understanding of these relationships. For this purpose, we used the ES-12 (1) to identify subgroups of adolescents with different profiles on the three *ES-12* subscales, and (2) to see how these profiles are associated with patterns of psychological health problems (disordered eating, non-suicidal self-injury, anxiety, and depression).

## Method

### Participants

The participants were identical to those from Sample 2 and 3 in the study by Foster et al. (2025). Altogether, these samples comprised 530 adolescents (259 girls, 262 boys, and 9 undisclosed or not identifying as either a girl or boy). All students were from grade 7 to 9 in public junior high school and their age ranged from 13 to 17 years, with a mean age of 14 years (*SD* = 0.89).

### Procedure

Students in public junior high school completed a digital survey using personal or school-provided laptops, tablets, or cellphones, with the survey link emailed to them by the researchers. Information about the project's aims and content, including details about confidentiality and the voluntary nature of participation, was sent to both students and their parents prior to data collection. This information emphasized that students were free to refrain from participating in the survey without providing reasons. Parents were informed that they could contact the project leader or class teacher to prohibit their child's participation in the survey. All participants provided digital consent to participate in the study before completing the survey.

Data collection took place during a designated lecture hour in the classroom. A clinically trained researcher and a research assistant administered the survey, while teachers were present to maintain order but did not participate in the administration process. Additionally, a clinically trained psychologist was available on-site, via phone or e-mail to address any iatrogenic effects or other problems and concerns that could arise during the survey or up to a week after data collection. Ethical approval was provided by the Swedish national ethics review board (registration numbers 2020-05885; 2021-06695-01; 2022-02093-02).

### Measures

*Embodiment* was assessed using the 12-item Embodiment Scale (*ES-12*), which contains 12 statements divided into three subscales; *Harmonious Body* (HB; e.g., “I feel at home in my body.”), *Disharmonious Body* (DB; e.g., “It happens that my body feels completely foreign to me.”), and *Body for Others* (BO; e.g., It is important to me what other people think about my appearance and physical characteristics.”). For a complete list of the items, see Foster et al. (2025; Table A5). The respondents rate the items as how often they have the corresponding experiences, from 1 to 5 (where 1 means “never” and 5 means “very often”). Cronbach’s alpha values were as follows: HB = .85, DB = .77, BO = .79, and for the total scale .88.

*Disordered eating* (DE) was assessed by the SCOFF questionnaire (Hansson et al., 2016; Morgan et al., 1999), which contains five questions concerning eating habits and attitudes toward weight and body shape, that are answered in a yes/no format (e.g., “Do you believe yourself to be fat when others say you are thin?”). A total score is computed as the number of questions that were given a positive answer.

*Depression and anxiety* were assessed using a 25-item version of Revised Children’s Anxiety and Depression Scale (RCADS-25; Chorpita et al., 2000; Ebesutani et al., 2012), which contains two subscales to measure anxiety and depression. The *Anxiety* subscale consists of 15 items (e.g., “I worry when I think I have done poorly at something”) and the *Depression* subscale consists of 10 items (e.g., “Nothing is much fun anymore”). Cronbach’s alpha values were .86 for the Anxiety subscale and .88 for the Depression subscale. *Non-suicidal self-injury* (NSSI) was assessed with a 9-item version of the Deliberate Self-Harm Inventory (DSHI-9r; Gratz, 2001; Lundh et al., 2011), where the respondents are asked to indicate how often they have deliberately injured themselves (e.g., by cutting, carving, or severely scratching themselves, or preventing wounds from healing) in the past 6 months. This is done on a scale from 0 (never) to 6 (more than five times), and a total score (range 0−54) is computed by summing all items. Cronbach’s alpha was .88.

### Cluster Analysis

Hierarchical cluster analysis was used to group all participants in two ways: (1) based on the profiles of their scores on the three subscales of *ES-12*, and (2) based on the profiles of their scores on the four measures of psychological health problems. The statistical package for pattern-oriented analyses in ROPstat (Vargha et al., 2015, 2016) was used at all steps of the procedure. First, the *Residue* module in ROPstat was used to identify and exclude multivariate outliers, defined as not having a “twin” within an Average Euclidean distance of 0.7. Second, the *Hierarchical* module was used, with Ward’s clustering method and a relocation procedure to improve the homogeneity of the clusters. The following criteria, taken from Bergman (1998) were used to identify the best cluster solution: (1) the number of clusters should not be expected to be less than five; (2) the size of the Explained Error Sum of Squares (EESS) for the chosen cluster solution should preferably not be less than 67%; (3) the homogeneity coefficient of each cluster should preferably be < 1; and (4) the cluster solution should be theoretically interpretable. Finally, using the *Validation* module in ROPstat, a data simulation was undertaken to verify that the explained ESS was higher than what could be expected on a random data set with the same general properties as the data set used in the real analysis.

To study the associations between the participants’ *ES-12* profiles and their profiles of psychological health problems, we cross-tabulated the two cluster solutions and significance-tested the difference between observed and expected frequencies for combination of categories with a two-tailed test by means of the *EXACON* module in ROPstat. Patterns that are significantly more common than expected are called *types*, and patterns that are significantly less common than expected are called *antitypes*.

## Results

Two separate hierarchical cluster analyses were carried out. First, a cluster analysis was carried out on the three *ES-12* variables, which led to the identification of five subgroups of adolescents with different *ES-12* profiles. Then, a second cluster analysis was carried out on four variables of psychological health problems, which led to the identification of five subgroups of adolescents with different profiles of psychological health.

### Cluster Analysis of the ES-12 profiles

**Figure 1:**
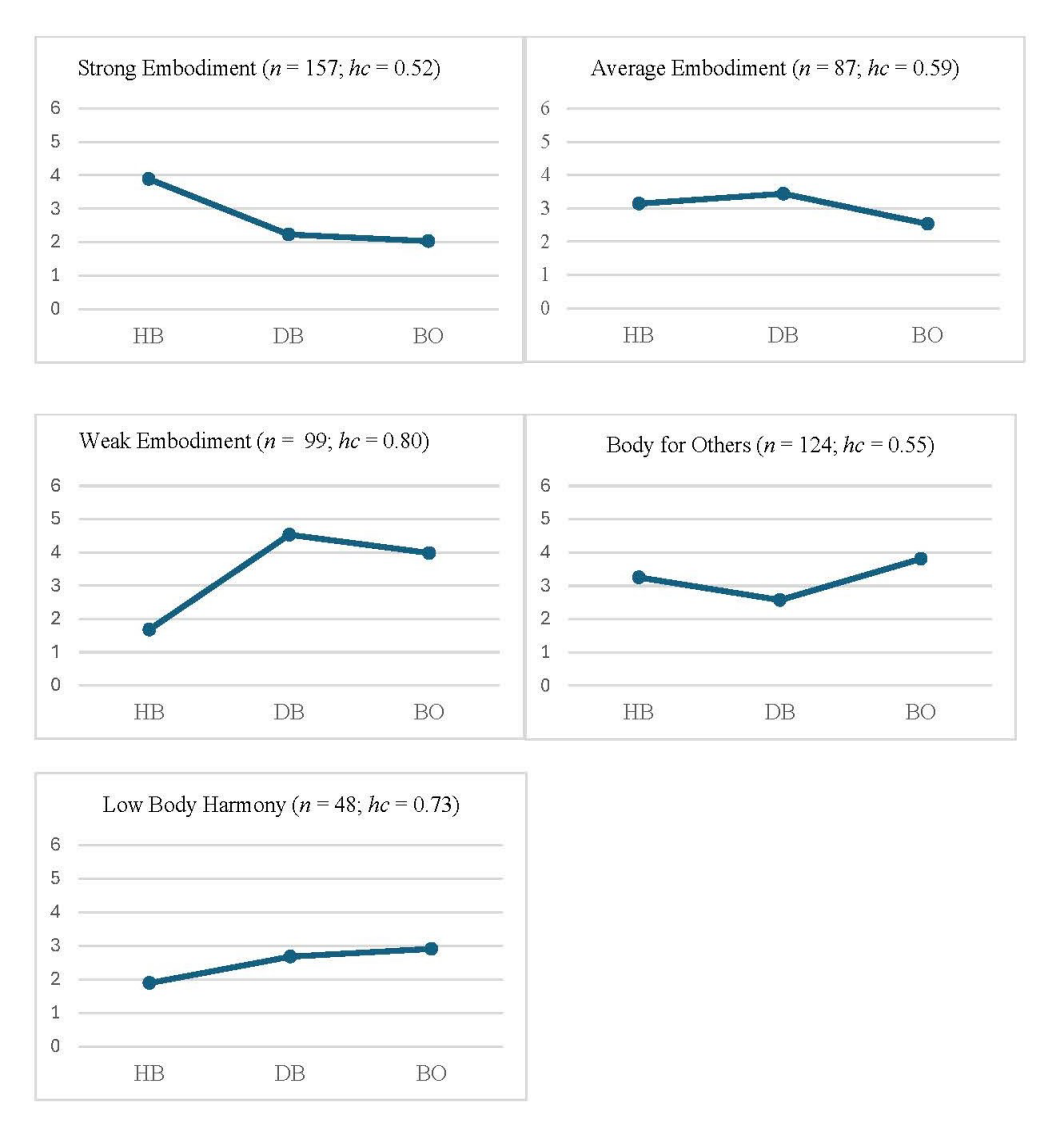
Profiles of the five ES-12 clusters, in terms of z+3-scores (where 3 corresponds to the sample mean on each ES-12 scale), with homogeneity coefficients (hc) for each cluster. HB = Harmonious Body; DB = Disharmonious Body; BO = Body for Others.

In total, 516 individuals had complete data on the *ES-12* and were included in the cluster analysis. One of them was identified as a multivariate outlier and was excluded, thereby leaving 515 individuals for the analysis. Based on Bergman’s (1998) criteria, a five-cluster solution was chosen which, after relocation, explained 69.7% of the ESS. The homogeneity coefficients for all clusters were <1 (ranging from 0.52 to 0.80; see Figure 1). Data simulation showed that the explained ESS was significantly higher than expected by chance (*p* < .001). Table 1 describes the clusters in terms of their unstandardized scores on the *ES-12* subscales, and Figure 1 shows the standardized scores.

**Table 1:** The Five Embodiment Clusters Described in Terms of Mean Values (and Standard Deviations) on the ES-12 Subscales.

	ES-12 subscale		
ES-12 Cluster	Harmonious Body	Disharmonious Body	Body for Others
Strong Embodiment (n =157)	4.26 (0.46)	1.24 (0.29)	2.14 (0.67)
Average Embodiment (n =87)	3.53 (0.53)	2.14 (0.37)	2.66 (0.63)
Weak Embodiment (n =99)	2.11 (0.56)	2.97 (0.58)	4.22 (0.57)
Body for Others (n =124)	3.64 (0.56)	1.49 (0.39)	4.03 (0.53)
Low Body Harmony (n = 48)	2.31 (0.54)	1.58 (0.40)	3.08 (0.77)

The *ES-12* clusters were named in accordance with the peaks (*z* >0.50) and troughs (*z* < -0.50) of their profiles. Thus, a large cluster, which contained 30.5% of the sample, was labelled *Strong Embodiment*, because it had a high score on HB (*z* = 0.89) and low scores on DB (*z* = -0.77) and BO (*z* = -0.97). A somewhat smaller cluster (16.9% of the sample) was labelled *Weak Embodiment*, as it had the opposite pattern of a low score on HB (*z* = -1.32) and high scores on DB (*z* = 1.53) and BO (*z* = 0.98). A third cluster (19.2% of the sample) had about average scores on all three subscales and was labelled *Average Embodiment*. Finally, there were two clusters that had diverging scores on only one of the subscales and that were labelled after that subscale. One of these (24.1% of the sample) was thus labelled *Body for Others*, as it showed a high score (*z* = 0.81) on BO and about average scores on the other two subscales; and the other (9.3% of the sample) was labelled *Low Body Harmony*, as it scored low on HB (*z* = -1.11) but around average on the other two subscales.

### Cluster Analysis of the psychological health profiles

**Figure 2:**
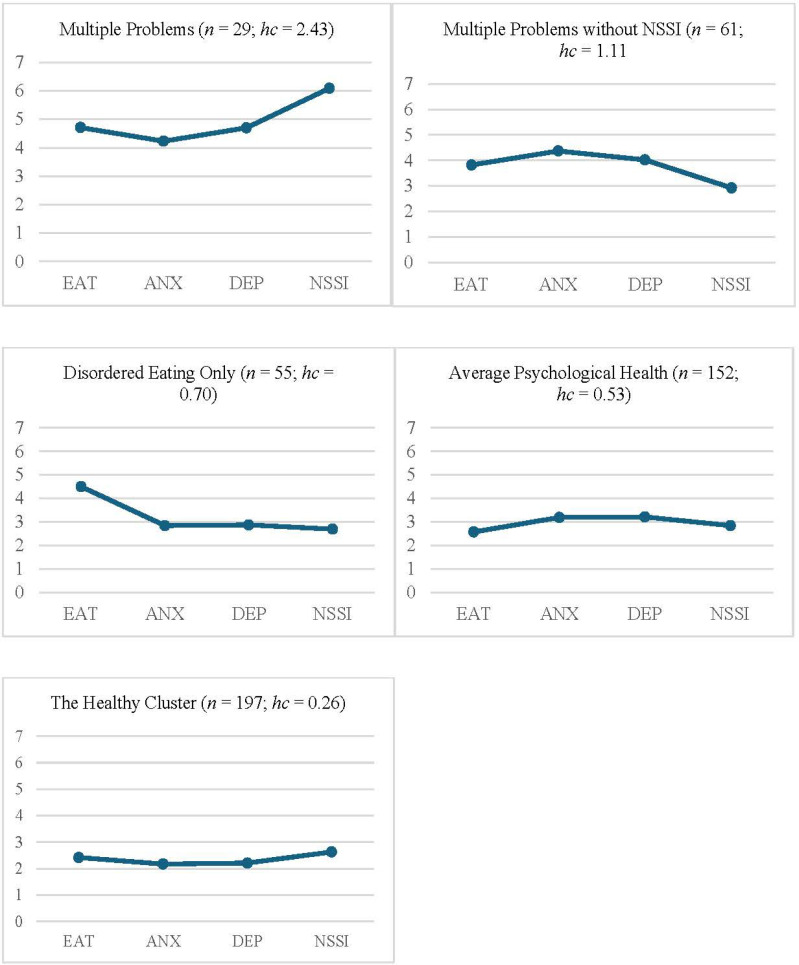
Profiles of the five psychological health clusters, in terms of z+3-scores (where 3 corresponds to the sample mean on each measure), with homogeneity coefficients (hc) for each cluster. EAT = Disordered eating, as measured by the SCOFF questionnare; ANX = Anxiety as measured by the RCADS-25; DEP = Depression as measured by the RCADS-25; NSSI = Non-suicidal self-injury as measured by the DSHI-9r.

A cluster analysis was also carried out on the four measures of psychological health problems: disordered eating as measured by the SCOFF questionnaire, symptoms of depression and anxiety as measured by the RCADS-25, and NSSI as measured by the DSHI-9r. In total, 502 individuals had complete data on these variables and were included in the cluster analysis. Four of them were identified as multivariate outliers and excluded, thereby leaving 498 individuals for the analysis. A five-cluster solution was chosen which, after relocation, explained 66,5% of the ESS. The homogeneity coefficients for the clusters ranged from 0.26 to 2.43. Data simulation showed that the explained ESS was significantly higher than expected by chance (*p* < .001). Table 2 describes the clusters in terms of their unstandardized scores on the psychological health variables, and Figure 2 shows the standardized scores.

**Table 2:** The Five Psychological Health Clusters Described in Terms of Mean Values (and Standard Deviations) on the four psychological health variables.

	Psychological Health Variables			
Psychological Health Clusters	DE	Anxiety	Depression	NSSI
Multiple Problems (n = 29)	2.55 (1.12)	21.93 (8.45)	19.07 (5.78)	31.38 (9.85)
Multiple Problems without NSSI (n = 61)	1.67 (1.08)	22.70 (5.15)	16.23 (3.89)	4.77 (4.56)
Disordered Eating Only (n = 55)	2.47 (0.74)	12.91 (5.30)	8.40 (2.99)	1.56 (3.28)
Average Psychological Health (n = 152)	0.30 (0.46)	13.39 (9.28)	10.15 (3.27)	2.20 (3.78)
The Healthy Cluster (n = 197)	0.13 (0.35)	5.77 (2.91)	4.55 (2.56)	0.54 (1.99)

*Note*. DE = Disordered Eating as measured by the SCOFF questionnaire; Anxiety and Depression was measured by the RCADS-25; NSSI = non-suicidal self-injury as measured by the Deliberate Self-Harm Inventory (DSHI-9r).

Again, the resulting five clusters were named in accordance with the peaks (z > 0.50) and troughs (z < -0.50) of their profiles. Thus, a small cluster (5.8% of the sample) was labelled *Multiple Problems*, because the adolescents in this subgroup had high scores on all four measures of psychological health problems. Another somewhat larger cluster (12.2% of the sample) was labelled *Multiple Problems without NSSI*, because the adolescents in this subgroup scored high on all psychological health problems except NSSI (z = 0.16). A third cluster (11.0% of the sample) was labelled *Disordered Eating Only,* as it had a high score only on the SCOFF questionnaire (z = 1.53) and scored around average on the other psychological health problems. A fourth large cluster (30.5% of the sample) was labelled *Average Psychological Health*, as it scored around the average on all four measures. Finally, the largest cluster (39.6% of the sample) was labelled the *Healthy* cluster, as it scored below average on all four psychological health problems.

### Cross-Tabulation of the ES-12 Clusters with the Psychological Health Clusters

To study associations between embodiment profiles and the profiles of psychological health problems, the five *ES-12* clusters were cross-tabulated with the five psychological health clusters (see Table 3). The EXACON module in ROPstat was used to identify types and antitypes, which are marked as ^T^ and ^A^ in the table. Because 25 tests were made, Bonferroni correction was used to set *p* < .05/25 = .002. As seen in Table 3, the *Strong Embodiment* profile was significantly over-represented among individuals with the *Healthy* profile (i.e., the combination of these two profiles was a *type*), and significantly under-represented among the individuals in the other four clusters (i.e., the combinations of a strong embodiment profile with these profiles were *antitypes*).

**Table 3:** Cross-Tabulation of the ES-12 Clusters with the Psychological Health Clusters. Observed Valued (Expected Values within Parentheses). Analysis of Types and Antitypes by EXACON.

	Psychological Health Clusters					
*ES-12 Clusters*	Multiple Problems	Multiple Problems without NSSI	Disordered Eating	Average Psychological Health	The Healthy Cluster	Total
Strong Embodiment	1 (8.9) ^A^	1 (18.3) ^A^	3 (16.5) ^A^	27 (45.3) ^A^	116 (59.0) ^T^	148
Average Embodiment	2 (5.2)	7 (10.7)	7 (9.6)	42 (26.3) ^T^	28 (34.3)	96
Weak Embodiment	22 (5.3) ^T^	37 (10.9) ^T^	12 (9.8)	16 (26.9)	1 (35.1) ^A^	88
Body for Others	2 (7.0)	8 (14.5)	23 (13.1) ^T^	41 (35.8)	43 (46.7)	117
Low Body Harmony	2 (2.7)	7 (5.6)	9 (5.0)	22 (13.8)	5 (17.9) ^A^	45
Total	29	60	54	148	193	484

A Antitype, i.e., the observed cell frequency is significantly less common than the expected (*p* <.002)

T Type, i.e., the observed cell frequency is significantly more common than the expected (*p* <.002)

Of most interest for the present study, however, are the three *ES-12* clusters with elevated scores on one or more *ES-12* subscales: *Weak Embodiment* (with high scores on all three *ES-12* subscales), *Body for Others* (with elevated scores only on the BO subscale), and *Low Body Harmony* (with low scores on HB). As seen in Table 3, the *Weak Embodiment* profile was significantly over-represented among the adolescents in the two multiple problem clusters, and significantly under-represented among the adolescents with the *Healthy* profile. The *Body for Others* profile was significantly over-represented among individuals with a *Disordered Eating Only* profile. Finally, the *Low Body Harmony* profile was under-represented among individuals with the *Healthy* profile.

## Discussion

There are three main findings of the present study. First, two subgroups of adolescents with opposite embodiment profiles were identified: one that was labeled *Strong Embodiment* which was clearly associated with psychological health, and another labeled *Weak Embodiment* which was equally clearly associated with multiple psychological health problems. These findings clearly align with theoretical frameworks that emphasize the importance of the concept of embodiment for the understanding of psychopathology (e.g., Fuchs & Schlimme, 2009).

Second, a subgroup of adolescents with high levels *only* on the dimension Body for Others was identified. Although these adolescents showed high levels of disordered eating, they showed no other signs of psychological health problems. These results are not consistent with theories (e.g., Stanghellini et al., 2012, 2019) which emphasize the central psychopathological significance of experiencing one’s body first and foremost as an object being looked at by others.

Third, the cluster analysis also identified a subgroup of adolescents that was labelled *Low Body Harmony*, because they showed low levels on the Harmonious Body dimension but average levels on the two other embodiment dimensions; this subgroup did not show any association with multiple psychological problems. This indicates that neither high levels of Body for Others nor low scores on Harmonious Body are sufficient in themselves for the development of multiple psychological health problems. These results are discussed in more detail below.

## Embodiment and Psychological Health

Five embodiment profiles were identified: *Strong Embodiment, Average Embodiment, Weak Embodiment, Body for Others,* and *Low Body Harmony*. The first three profiles merely represent different degrees of embodiment and thereby contribute little beyond what a purely variable-oriented approach would reveal. The two latter, however, illustrate how a person-oriented approach can contribute with new findings beyond those offered by a variable-oriented approach.

The cluster analysis of the psychological health measures (disordered eating, anxiety, depression, and NSSI) similarly adds findings beyond what is shown by a pure variable-oriented approach. Five clusters were identified: *Multiple Problems, Multiple Problems without NSSI, Disordered Eating Only, Average Psychological Health*, and a *Healthy* profile. Four of these can be arranged along a single dimension of psychological health, from (1) the *Multiple Problems* cluster, which is a small cluster with less than 6% of the adolescents, who show very high scores (*z* > 3) on NSSI and high scores (*z* > 1) also on the other three problem dimensions; (2) a somewhat larger cluster (*Multiple Problems without NSSI*) with 12,2% of the adolescents, who show average scores on NSSI, and *z*-scores > 1 on anxiety and depression, and (3) a large cluster of adolescents (more than 30%) with *Average Psychological Health*, to (4) an even larger *Healthy* cluster (containing almost 40% of the adolescents). The fifth cluster, *Disordered Eating Only* (11% of the adolescents), however, is difficult to fit into a one-dimensional psychological health dimension, as it combines elevated levels (in fact, a very high score of *z* = 1.5) on one of the psychological health problems (disordered eating) with scores slightly *below* average on the other three psychological health problems (with *z*-scores ranging from -.13 to -.31 on NSSI, anxiety and depression). This clearly motivates the labeling of this cluster as *Disordered Eating Only* and raises the question of how to understand this kind of pattern.

It is noteworthy that the adolescents with the *Disordered Eating Only* profile scored almost equally high as those with the *Multiple Problems* profile on disordered eating (*z*-scores of 1.5 as compared with 1.71), despite their very different scores on depression, anxiety and NSSI. In combination with previous research, these results for the *Multiple Problems* cluster suggest that adolescents in this cluster may show clinically relevant forms of disordered eating, whereas those in the *Disordered Eating Only* cluster probably suffer primarily from non-clinical forms of disordered eating. The size of the subgroup with the *Multiple Problems* profile (slightly

below 6% of the sample) is relatively consistent with the life-time prevalence of eating disorders, which is reported to be around 5% (e.g., Treasure et al., 2010), and with evidence that eating disorders have high comorbidity with depression and anxiety disorders. Blinder et al. (2006), for example, found that 94% of female inpatients with primary DSM-IV diagnoses of eating disorders (anorexia, bulimia, and eating disorder not otherwise specified) showed comorbid mood disorders (mainly unipolar depression), and that 56% of them showed anxiety disorders. Adding to this picture, a systematic review and meta-analysis by Meier et al. (2024) showed that around one third of children and adolescents with eating disorders have engaged in NSSI at least once in their life. Altogether this suggests that the adolescents with the *Multiple Problems* profile may represent a clinically relevant group, whereas those with the *Disordered Eating Only* profile show little evidence of psychopathology.

At the same time, these two subgroups were associated with very different embodiment profiles. Whereas the adolescents with the *Multiple Problems* profile showed a pattern of *Weak Embodiment* generally, those with the *Disordered Eating Only* profile showed high levels on only one of the embodiment dimensions: Body for Others. These results are of special interest in relation to theories about the psychopathological significance of different aspects of embodiment.

## Body for Others and Disordered Eating

Adolescents with the embodiment profile *Body for Other*s showed elevated scores only on the subscale Body for Others (BO). It is interesting to note that, although this embodiment profile was significantly over-represented among individuals with a *Disordered Eating Only* profile, it showed no tendency to be over-represented among the adolescents with the two *Multiple Problem* profiles – as seen in Table 3, if anything, it rather tended to be under-represented among those with *Multiple Problem* profiles. This suggests that the large group of adolescents with the *Body for Others* profile (24% of the entire sample) are not typically in the risk zone for any severe form of psychopathology. Although they attach large importance to how others view their body, and tend to engage in problematic eating behaviors, they show no other signs of disturbed embodiment or psychological ill-health. First, they scored slightly above average on *Harmonious Body* and slightly below average on *Disharmonious Body*. Second, they showed no tendency to be over-represented among the adolescents with the patterns of *Multiple Problems* and *Multiple Problems without NSSI*.

Interestingly, the adolescents with the *Body for Others* profile and those with the *Weak Embodiment* profile scored almost identically high on the BO subscale (*z* = 0.98 and *z* = 0.91, respectively), and yet they were associated with widely different profiles of psychological health problems. This provides an illustrative example of how *the same value on one specific variable can mean very different things* in a more holistic perspective, as being part of different “Gestalts” (e.g., Bergman & Andersson, 2010). When a high score on BO is part of an embodiment profile that involves low levels of body harmony and high levels of body disharmony (as shown by the adolescents with the *Weak embodiment* profile), they mean one thing (increased risk for multiple psychological problems); but when the same score on BO is part of another embodiment profile (as shown by the adolescents with the *Body for Others* profile), they mean something quite different (no evident risk for multiple psychological problems).

## Low Body Harmony

The embodiment profile *Low Body Harmony* illustrates something partly similar. This profile, which combined low scores on HB with average scores on DB and BO, showed no over-representation among adolescents with multiple problem profiles, or among adolescents with disordered eating. It was, however, under-represented among adolescents with a *Healthy* profile. Although this suggests that a deviating score only on this subscale scales is probably not a sufficient reason for concern, the question remains what this kind of pattern means for adolescents’ well-being in a developmental perspective. For example, could it be that this kind of profile represents a risk factor for future negative developments?

As with all the present results, this points to the need for longitudinal studies of the development of adolescents with the different embodiment profiles that were identified in the present study. Although the present results are generally consistent with theoretical models that depict embodiment disturbances as causal factors behind various forms of psychopathology (e.g., Fuchs & Schlimme, 2009), they are equally consistent with the possibility that disturbances of embodiment represent a symptom of psychological ill-health – and, of course, with the possibility that there obtains a circular relationship between the two, so that they reciprocally reinforce each other. The testing of these theoretical possibilities requires longitudinal studies with repeated measurement during adolescence to establish the timeline for changes in embodiment and in psychological health.

## Limitations and Future Research

As already emphasized, a main limitation of the present study is that the data are merely cross-sectional and can therefore say nothing about the development of embodiment profiles and psychological health. This means that the results do not allow for any conclusions about causality or risk factors in a developmental perspective. Stronger conclusions must wait until longitudinal data are available for analysis. Another limitation of the study is that the sample consists solely of young community adolescents, and that the results cannot be generalized to more diverse or clinically relevant populations. To address this limitation, future studies should include clinical samples to better understand how embodiment profiles and psychological health manifest in populations with more pronounced or diagnosed conditions.

## Data Availability

The data that support the findings of this study are available on request from the corresponding author. The data are not publicly available due to privacy or ethical restrictions.

## Action editor

Lars R. Bergman served as action editor for this article.

## Open access

This article is distributed under the terms of the Creative Commons Attribution 4.0 International License (http://creativecommons.org/licenses/by/4.0/), which permits unrestricted use, distribution, and reproduction in any medium, provided you give appropriate credit to the original author(s) and the source, provide a link to the Creative Commons license, and indicate if changes were made.
